# The rise and fall of diseases: reflections on the history of population health in Europe since ca. 1700

**DOI:** 10.1007/s10654-021-00719-7

**Published:** 2021-02-20

**Authors:** Johan P. Mackenbach

**Affiliations:** Department of Public Health, Erasmus MC, P.O. Box 2040, 3000 CA Rotterdam, The Netherlands

**Keywords:** History, Population health, Diseases, Epidemiological transition

## Abstract

This essay explores the amazing phenomenon that in Europe since ca. 1700 most diseases have shown a pattern of 'rise-and-fall'. It argues that the rise of so many diseases indicates that their ultimate cause is not to be sought within the body, but in the interaction between humans and their environment. In their tireless pursuit of a better life, Europeans have constantly engaged in new activities which exposed them to new health risks, at a pace that evolution could not keep up with. Fortunately, most diseases have also declined again, mainly as a result of human interventions, in the form of public health interventions or improvements in medical care. The virtually continuous succession of diseases starting to fall in the 18th, 19th and 20th centuries suggests that the concept of an “epidemiological transition” has limited usefulness.

## Introduction

The rise of life expectancy is one of the most important, if not the most important, event in human history. In many European countries life expectancy trends can be traced back to the nineteenth century, and show that since then life expectancy has doubled. For example, in the Netherlands average life expectancy at birth rose from about 40 years in the 1860s to about 80 years in the 2010s. And not only do we live longer, we also live much longer in good health than our ancestors in the nineteenth century [1].

The explanation of the tremendous decline in mortality underlying this increase in life expectancy is complex, and is partly captured by the theory of the “epidemiological transition” which was proposed by Abdul Omran (1925–1999) in the early 1970s. He pointed out that we live longer because—to put it simply—we have exchanged infectious diseases, which take their toll at a young age, for chronic diseases such as cardiovascular disease and cancer, from which we mainly die at an older age [2].

This theory has since been criticized and expanded, but it is certainly true that over the last few centuries there has been a remarkable succession of diseases that rose and then went down again. In a kind of ‘procession of Echternach’ Europeans often took a few steps forward, by overcoming some diseases, and then a step back again, when a new disease emerged. Complete setbacks, such as temporary declines in life expectancy, were rare, but at all times the speed of progress in population health depended on the balance between declining diseases on the one hand, and rising diseases on the other hand [3] (pp. 45–48).

This amazing phenomenon of ‘rise-and-fall’ of diseases has often been noted, but has only rarely been discussed systematically. In this essay I will first illustrate that many diseases have shown a pattern of rise-and-fall, and then discuss some implications of this generalized phenomenon. What does the fact that so many diseases have become more common, reveal about their causes? What explains the fact that most diseases have, after a longer or shorter period, retreated? And what are the implications of this phenomenon for the theory of the epidemiological transition? I will end with a few words on how the current pandemic of COVID-19 fits into this story^1^.

## A generalized phenomenon

That human diseases tend to occur in a pattern of rise-and-fall has been noted by, among others, René Dubos (1901–1982) [4] and David Barker (1938–2013) [5], but their observations were largely anecdotal. Also, changes in disease classification imply that not all apparent rises-and-falls are real [6]. Nevertheless, it has been possible to reconstruct long-term trends in a range of European countries for no less than 43 health problems, and the available data show that at least 34 out of these 43 have occurred in a pattern of rise-and-fall (Table 1) [3].

**Table 1:** Rise and fall of diseases in Europe, ordered by timing of decline. *Source*: ref. [3], where full references to timing of rise and fall can be found

Health problem	Rise and fall?		Start of rise^a^	Start of fall^b^
War	Rise and fall		before 4300 BCE	Sixteenth century^c^
Homicide	Fall only		N/A	Sixteenth–seventeenth century
Plague^d^	Rise and fall		1347	Seventeenth century
Typhus	Rise and fall		Late fifteenth century	Seventeenth century?
Famine	Rise and fall		6500 BCE^e^	Eighteenth century
Smallpox	Rise and fall		Sixth century	Eighteenth century
Malaria	Rise and fall		Sixteenth century	Eighteenth century
Cholera	Rise and fall		1829–1837	1846–1860
Three intestinal infections^f^	Rise and fall		6500 BCE^e^	Mid-nineteenth century
Tuberculosis	Rise and fall		Eighteenth century	Mid-nineteenth century
Puerperal fever	Rise and fall		Eighteenth century	Mid-nineteenth century
Infant mortality	Fall only		N/A	Late eighteenth–late nineteenth century
Four childhood infections^g^	Rise and fall		Eighteenth century	Late nineteenth century
Pellagra^h^	Rise and fall		Eighteenth century	Late nineteenth century
Rickets	Rise and fall		Seventeenth century	Late nineteenth century
Syphilis	Rise and fall		Late fifteenth century	Early twentieth century
Pneumoconiosis	Rise and fall		Nineteenth century	Early twentieth century
Pneumonia	Fall only		N/A	Early twentieth century
Stomach cancer	Rise and fall		Nineteenth century?	Early twentieth century
Influenza	Rise and fall		Sixteenth century	1918–1919
Goitre	Fall only		N/A	1920s
Peptic ulcer	Rise and fall		Late nineteenth century	1930s-1940s
Appendicitis	Rise and fall		Late nineteenth century	1930s–1940s
Still-births	Fall only		N/A	1940s
Suicide	Rise and fall		Eighteenth century	1920s–1980s
Ischemic heart disease	Rise and fall		Early twentieth century	1970s
Cerebrovascular disease^i^	Rise and fall		Early twentieth century	1970s
Colorectal cancer	Rise and fall		Early twentieth century?	1970s
Road traffic injuries	Rise and fall		Early twentieth century	1970s
Lung cancer	Rise and fall		1930s	1970s–1980s^j^
Liver cirrhosis^k^	Rise and fall		1950s	1970s–2000
Breast cancer	Rise and fall		Late nineteenth century?	1980s
Prostate cancer	Rise and fall		First half twentieth century?	1980s–1990s
Aids	Rise and fall		Early 1980s	Mid-1990s
Mesothelioma	Rise only		1970s	N/A
Diabetes mellitus type II	Rise only		Mid-twentieth century	N/A
Dementia^l^	Rise only		1970s	N/A
Depression	Trends unknown		N/A	N/A

*N/A* not applicable

^a^Approximate start of rise in Europe

^b^Approximate start of fall (or peak-year) for North-western Europe only

^c^Peak in frequency of war (not in war deaths)

^d^Second pandemic only

^e^In Europe, the Neolithic or first Agricultural revolution started in the Aegean around 6500 BCE

^f^Dysentery, typhoid fever, paratyphoid

^g^Scarlet fever, measles, whooping cough, diphtheria

^h^Timing for pellagra is for Southern and South-eastern Europe

^i^Trend for ischemic stroke only

^j^Decline in men only

^k^There may have been another ‘rise-and’fall’ cycle in the nineteenth century

^l^Trend for mortality; no evidence for rise in age-adjusted prevalence

From war victims to AIDS, from plague to puerperal fever, and from appendicitis to breast cancer: if one looks back far enough in time, it can be seen that most diseases whose history we know have had a spectacular rise and an equally spectacular fall. Some diseases, such as AIDS, apparently started from zero, whereas others, such as breast and other cancers, probably had always occurred at relatively low frequencies [7] (Ch. 14), but at some point in time started to rise importantly.

The rise sometimes started very long ago: for example, some infectious diseases already rose when humans became farmers or started living together in cities. Plague (i.e., what was actually the second pandemic) arrived in Europe in 1347, and syphilis in the 1490s, both as a result of long-distance travel. But almost always there also was a turning point. Some diseases, such as plague and typhus, started to decline as early as the seventeenth or eighteenth centuries. Others, such as puerperal fever and syphilis, started to decline in the nineteenth or first half of the twentieth century, while others still, such as ischemic heart disease and liver cirrhosis, only started to decline in the second half of the twentieth century. There are also diseases that are still on the rise but hopefully will go down in the future, such as diabetes and dementia.

Let me briefly describe a few examples. Malaria is a disease that rose as an accompaniment to agriculture. It was once endemic to Europe, even above the Arctic Circle. But from the eighteenth century onwards, the disease was pushed back, first in Northern and Western Europe, later also in the South and East. This decline was the result of drainage of swamps, better nutrition, use of quinine, and later also of targeted control campaigns, including with DDT [8].

Europe’s industrialization and urbanization then led to the rise of other diseases, including tuberculosis. The history of this disease has become famous through the work of Thomas McKeown (1912–1988), who showed that the decline of tuberculosis in England had started long before the introduction of antibiotics. He concluded that medical care has been less important to the increase in life expectancy than many people thought in his day [9].

However, we now know a lot more and can follow the rise-and-fall of tuberculosis in several European countries. The decline was not mainly due to better nutrition, as McKeown thought, but largely due to human intervention, in the form of isolating patients, improvements in housing and working conditions, pasteurization of milk, and also the use of antibiotics, particularly in Southern and Eastern Europe [10, 11].

Eventually, not only tuberculosis, but also the other diseases of industrialization went into decline, but were exchanged for new health problems, such as ischemic heart disease, diabetes, various forms of cancer, and road traffic injuries. However, just like malaria and tuberculosis, these are also ‘diseases of progress’.

Cancer is a good example. Many cancers have increased dramatically over the course of the twentieth century due to changes in living conditions and behaviour, as a result of what we usually regard as economic and social progress. Think of the large-scale industrial production of what is good for us, but also of cigarettes, alcoholic drinks, and asbestos, which cause cancer. And think of postponing having children, which creates time for following higher education, but also increases the risk of breast cancer. Fortunately, cancer has also passed its turning point, at least in terms of mortality, thanks in part to earlier detection and better treatment [12].

## Fundamental causes of disease

It is amazing that so many diseases have shown a pattern of rise-and-fall. Why, in the course of European history, have so many diseases become more common? And why have most of these diseases subsequently declined, or at least become less lethal?

In my opinion, too little thought has been given to such fundamental questions, except perhaps by René Dubos, who already wrote in the 1960s that most diseases are the result of a mismatch between people and the environment. For example, in his view “[t]he diseases characteristic of highly industrialized and urbanized societies are, to a large extent, the manifestations of the effects of new environmental forces to which man has not had a chance to become adapted” [4] (p. 367).

I too think that the emergence of diseases indicates that their ultimate cause is not to be sought within the body. Diseases do not come ‘from within’, because on a time-scale of centuries spontaneous (e.g., genetically driven) changes in the structure and functioning of the body are simply impossible. Instead, diseases arise from the interaction between humans and their environment [13].

Let me add a few tiny nuances to this bold statement. There are certainly diseases that are primarily due to genetic defects, without much influence from the environment, such as Huntington’s disease and Down’s syndrome—but these are relatively rare [14]. An endogenous cause like the inevitable failure of our body in old age is certainly becoming more important as we get more and more exogenous causes under control [15]—but epidemiological research shows that the vast majority of cases of disease can still be attributed to exogenous causes. This was famously demonstrated by Doll and Peto for cancer in the United States [16], and has since been demonstrated by the Global Burden of Disease study for a much wider range of diseases, using estimates of population-attributable fractions synthesized from risk factor data [17]. Knowledge of what happens inside the body is certainly of great importance, for example for finding a treatment—but if we want to combat diseases fundamentally, we have to go out into the wide world.

I will even take it a step further: in a sense, Europeans have been making their own diseases, because the rise of diseases was almost always linked to changes in human behaviour. Malaria, tuberculosis and cancer, just discussed, were clear examples, and there are many more. Long-distance trade brought plague and cholera from Asia, and syphilis from America. Urbanization massively increased not only tuberculosis, but also typhoid fever, diphtheria, measles and many other infectious diseases. Industrialization brought prosperity, but also air pollution, and the opportunity to eat more than is good for us [18].

And then one wonders: why has this happened over and over and over again? I think that this was because, in their tireless pursuit of a better life, Europeans constantly engaged in new activities which exposed them to new health risks, at a pace that evolution could not keep up with. In order to improve their living conditions, they undertook long journeys, started living together in larger groups, adopted new modes of production, and only later did it become clear that some of these changes were harmful to health.

This was not always as innocent as it seems. The pursuit of better living conditions and better health was littered with conflicting interests, because the activities that led to the rise of disease usually also generated money. This was already the case with the long-distance trade that brought plague and cholera to Europe [19], and it still is, as the fight against tobacco and the tobacco industry shows.

The rise of the cigarette—and with it the rise of lung cancer and other smoking-related diseases—encapsulates many paradoxical aspects of economic and social progress. Industrial production, increased purchasing power, a ‘modern’ way of life, women’s emancipation—it is all nice, but it has also enabled the large-scale distribution of the cigarette. Up to a point, the accompanying rise of lung cancer was perhaps a matter of bad luck. Ultimately, however, commercial interests ensured that cigarette sales continued, and still continue, long after the adverse health effects were established [20].

Fortunately, European history over the last few centuries shows that most diseases also declined again. Sometimes this was due to the self-limiting nature of a disease, as with some infectious diseases that became less deadly over time as the milder variants spread more successfully. But usually that decline was also “man-made”.

For most of the diseases that have declined over time, humans themselves had a hand in it. The pursuit of better living conditions usually resulted in fewer health risks, on balance and in the long term. This sometimes occurred unintentionally, for example when humans became less susceptible to infectious diseases through better nutrition. But more often this was the result of conscious intervention, when humans used part of the newly acquired prosperity for investments in a healthier environment, in prevention and medical care, and in scientific research into the causes of disease [3, pp. 281–284].

The good news is that the time-scale on which rise-and-fall of disease manifested itself has become shorter and shorter, as human intervention to control disease has become increasingly effective. The record-holder is the AIDS epidemic. Mortality from AIDS peaked in many European countries as early as the first half of the 1990s, less than 15 years after the epidemic started. This rapid turnaround, eventually all over Europe, was possible thanks to rapid investments in research and rapid application of research results in behaviour, prevention and medical care [21].

## Public health and other determinants

Squinting through the eye-lashes, interventions to push back disease can be seen to fall into three groups: non-targeted improvement of general living conditions, targeted elimination of specific health risks, and even more targeted slowing down of disease processes. Of these, the first two have been the only options for centuries, as medicine did not yet have effective treatment options. This implies that measures outside of health care proper, i.e., public health interventions, had to do the job.

And that is what happened, even as early as the seventeenth and eighteenth centuries [22], but it was in the nineteenth century that public health took on a recognizable face in the fight against cholera. Even before it became clear that cholera was spreading via polluted drinking water, and based on a limited understanding of the environmental causes of the disease, piped drinking water and sewers were chosen as the solution. These were constructed first in North-western Europe, but eventually these measures were also taken in Southern and Eastern Europe—a delay that is also clearly reflected in a delay in the decline of mortality from cholera [23].

It is in the confrontation with cholera that public health developed its paradigmatic approach, which can be summarized in three axioms. (1) Most diseases arise in the interaction between people and the environment, (2) so we can and must try to radically remove their causes, and (3) this can only be done if we take collective responsibility for these measures, i.e., if the government takes the lead [24].

Public health has played a decisive role in the decline of many diseases, not only of plague, smallpox, typhus and cholera, but also of pneumoconiosis, lung cancer, cirrhosis of the liver, road traffic injuries and many other health problems. Over the last three centuries, public health clearly wins out over other forms of human intervention [3] (pp. 284–287).

Over time, however, public health has been joined by better medical care. Since McKeown, not only has it become clear that antibiotics have played a role in the decline in mortality from infectious diseases, but also that it has played a role in other diseases. Medical treatments have contributed to the decline of many diseases, and have even played a decisive role in the decline of puerperal fever, stillbirths, appendicitis, stroke, and several others [25].

One of the best-known examples of the rise-and-fall of disease is ischemic heart disease. Evidence of atherosclerosis has been found in Egyptian mummies, suggesting that ischemic heart disease was probably not a ‘new’ disease [7] (Ch. 5), but it was rare before the end of the nineteenth century. Epitomizing the idea of a ‘disease of progress’, it rose quickly in the twentieth century, due to increased prosperity, more abundant diets, less exercise, and the epidemic of cigarette smoking. But its rise has turned into a spectacular decline, which has now spread all over Europe. This success has, as always, many fathers, but research has shown that both prevention and treatment, and both public health and medical care, have played an important role, probably in roughly equal proportions [26].

Improvements in public health have eventually taken place across Europe, but with wide variations in timing and pace. For three centuries, Northern and Western Europe have been systematically at the forefront, and Eastern Europe (despite some remarkable successes under communism in the middle of the twentieth century) in the rear-guard. This indicates the existence of structural factors that have promoted or hindered effective intervention [3] (pp. 291–323).

The first and best known is economic development: prosperity and life expectancy usually go hand in hand, because prosperity is an important pre-condition for health. North-western Europe has also been at the forefront of economic development for three centuries, which partly explains why population health has been better there than elsewhere [27].

But some countries are better at converting prosperity into health than others. The second structural condition for health is an effective state. The modern state apparatus is necessary to take collective measures to protect health, to direct an increasingly complex health care system, to distribute wealth in such a way that everyone’s health can benefit from it, et cetera [28].

But neither economic nor political factors have been the driving force behind the massive improvements in public health over the centuries. Without the emergence of rational thinking, in the form of the “Enlightenment”, economic and political conditions would not have changed for the better. That was the real driving force behind this history. It is thanks to the spread of a more rational way of thinking that agricultural methods were improved, the steam-engine has been invented, the state received support from a professional civil service, and that the spread of cholera through polluted drinking water has been established. And North-western Europe has always been at the forefront of the development of rational thinking as well [29].

## The epidemiological transition theory

Although the notion of an “epidemiological transition” has been widely accepted in the demographic and epidemiologic literatures, it has also become clear that Omran’s picture of these long-term changes in population health is not entirely accurate. For example, he characterized the shift in disease patterns as one from “pandemics of infectious diseases” to “degenerative and man-made diseases”. However, the vaguely moralistic heading of “degenerative and man-made diseases” is not an adequate label for the causes of death that have replaced the infectious diseases [30].

Although cardiovascular diseases and neoplasms are diseases of middle and old age, we now know that they are not primarily caused by age-related biological processes of “degeneration”, but are predominantly exogenously caused, just like the infectious diseases that dominated the cause-of-death pattern in previous centuries. Also, while many of these exogenous causes, such as smoking and excessive alcohol consumption and occupational exposures, are “man-made”, so were the living conditions and habits which promoted the transmission of infectious agents.

More importantly, now that more time has elapsed we can see more clearly what happened during the twentieth century, and we now also know what happened after Omran published his theory in the early 1970s. Coincidentally, just when he published his theory, a renewed decline of mortality started in many high-income countries. A reversal in the trend of ischemic heart disease was one of its main drivers, but declines in other causes of death such as road traffic injuries also played a role. This renewed decline has sometimes been referred to as a fourth stage of the epidemiologic transition, labelled the “age of delayed degenerative diseases” (because it was mistakenly thought that this did not entail an elimination of mortality from heart disease, but only a delay to older ages) [31].

Another surprise was that it soon appeared that infectious diseases had not been eliminated, but on the contrary seemed to return with a vengeance. After 1970, new infectious diseases emerged (such as legionnaire’s disease and AIDS), some old diseases re-emerged as a result of lapses in infectious disease control (such as tuberculosis and whooping cough), and some other infectious diseases became more difficult to control because of antibiotic resistance (such as hospital-acquired staphylococcal infections). Adding to the confusion, it was proposed to either label this a fifth stage of the epidemiological transition, or to consider this an entirely new epidemiological transition [32].

What this really demonstrates is that the long-term improvement in population health that the epidemiological transition tries to capture, was and still is a very heterogeneous phenomenon. It consisted of sequentially ‘falling’ diseases, with an emphasis on declines for some infectious diseases in one period, an emphasis on declines for some other infectious diseases in another period, and some emphasis on declines for cardiovascular diseases in still another period, but without clear-cut differences by type of disease, however defined.

Actually, the ‘telescope of history’ makes us see the “epidemiological transition” in a different perspective. The last decades of the nineteenth century and the first half of the twentieth century were certainly special, in the sense that many important causes of mortality declined rapidly and simultaneously. Yet, the absence of natural dividing-lines with earlier and later periods, and the virtually continuous succession of diseases starting to fall in the seventeenth, eighteenth, nineteenth and twentieth centuries (Table 1), suggest that the concept of an “epidemiological transition” has limited usefulness.

##  COVID-19

This is what one sees when studying long-term trends in population health. How does the COVID-19 pandemic fit into this picture? At the time of writing (October 2020) it is, of course, still early days but it is obvious that COVID-19 is once again a ‘disease of progress’, caused by a virus that effectively abuses our modern way of life. Intensive exploitation of the animal world allowed it to jump from bats to humans somewhere in Asia. Thanks to urbanization, it could easily spread through exhaled air. Thanks to globalization, it was able to flood the world within weeks [33].

In many respects, COVID-19 resembles influenza. Influenza came to Europe in the sixteenth century and over the following centuries caused several major epidemics with hundreds of thousands of deaths, spreading faster and faster over time thanks to steam-ships and steam-trains, and later air-planes [34]. The ‘Spanish’ flu of 1918 was the first influenza pandemic in which an attempt was made to contain its spread, but it still caused enormous excess deaths in many European countries. Subsequent influenza pandemics have caused much less health damage, and the pandemics of 1957 and 1968 can often hardly be seen in the all-cause mortality trends [35].

The death toll of the COVID-19 pandemic has so far been relatively limited. For example, in the first half of 2020 it caused about 10,000 extra deaths in the Netherlands (https://www.cbs.nl/nl-nl/nieuws/2020/40/10-duizend-coronadoden-tijdens-eerste-golf-van-de-pandemie). That is 10,000 too many, but if it stops at that, it will only leave a small spike in the historical time series. Because the deaths were mainly among elderly people with serious health problems, the number of years of life lost was relatively limited too.

That the health damage did not reach apocalyptic proportions was probably partly due to the drastic countermeasures. It may therefore well be that the historically unique feature of the COVID-19 pandemic will turn out not to be the large number of victims, but the sheer scale of the countermeasures. Never before in recent history have entire societies been partially shut down to contain a pandemic, and the sacrifices that had to be made were unprecedented.

Social isolation, closed schools, and scaled down health care services count among these sacrifices, and so does the economic damage of the ‘lockdowns’ which may cause rising unemployment in the years to come, despite huge financial support measures. One of the most striking features of this episode is that governments all around Europe—and beyond—have shown an astoundingly large ‘willingness-to-pay’ to avert the threat of a higher number of deaths. The COVID-19 pandemic thus also illustrates the continued importance of the state for health protection.

What will future generations think of this? We can only speculate, but I expect them to admire our decisiveness and solidarity, and at the same time to look back with gritted teeth, if only because this pandemic could have been avoided if we had heeded previous warnings. We knew that this could happen one day, for example after the SARS epidemic of 2002, but radical public health measures were not taken.

We could have done more to prevent virus jumping from wild animals to humans by closing ‘wet markets’ in Asia. We could have done more to prevent spreading, through better warning systems and faster route blocking. We could have prepared better, by devising less damaging lockdown scenarios, and by stocking protective equipment. And we could have strengthened the public health infrastructure instead of scaling it down, as happened after the 2008 financial crisis [36].

It is therefore to be hoped that the enormous ‘willingness-to-pay’ during the pandemic will also apply to measures to prevent the next pandemic [37].

## Footnotes

1 This essay is an edited version of my valedictory lecture as Professor of Public Health at Erasmus MC, Rotterdam, the Netherlands (https://youtu.be/364GyURJbw0). Most of the scientific underpinnings of the lecture can be found in my recent book, “A history of population health; rise and fall of disease in Europe” (Leiden & Boston: Brill | Rodopi, 2020). This open access publication, which contains extensive references, can be found at: https://brill.com/view/title/57111.
